# 3D quantification of wall shear stress using finite-element interpolations from 4D flow MR data in the Thoracic Aorta

**DOI:** 10.1186/1532-429X-16-S1-P356

**Published:** 2014-01-16

**Authors:** Julio A Sotelo, Jesus Urbina, Cristian Tejos, Israel Valverde, Daniel E Hurtado, Sergio Uribe

**Affiliations:** 1 Biomedical Imaging Center, Pontificia Universidad Católica de Chile, Santiago, Region Metropolitana, Chile; 2 Electrical Engineering, Pontificia Universidad Católica de Chile, Santiago, Region Metropolitana, Chile; 3 Radiology, School of Medicine, Pontificia Universidad Católica de Chile, Santiago, Region Metropolitana, Chile; 4 Pediatric Cardiology Unit, Hospital Universitario Virgen del Rocío, Sevilla, Spain; 5 Cardiovascular Physiopathology Laboratory, Biomedicine Institute of Seville, Hospital Universitario Virgen del Rocío, Sevilla, Spain; 6 Structural Engineering, Pontificia Universidad Católica de Chile, Santiago, Region Metropolitana, Chile; 7 Biomedical Engineering Group, Pontificia Universidad Católica de Chile, Santiago, Region Metropolitana, Chile

## Background

Actual methods to quantify wall shear stress (WSS) are performed on reformatted 2D planes from 4D flow data sets. This approach has the inherit limitation that only a few planes are analyzed on specific locations of the aorta, even though the full 3D velocity field is usually available. Another problem with this approach is that the process of locating 2D planes manually is dependent on the user and may lead to results that have low reproducibility. These problems can be circumvented by calculating the WSS in 3D directly. A few methods based on computational fluid dynamics (CFD) have been proposed to obtain 3D WSS. These methods use realistic vascular geometries extracted from MR data, however, assumptions are made on the properties of the walls and on flow velocity profiles that may not be fully realistic. In this work, we propose a novel methodology based on finite-element (FE) interpolations to compute the 3D WSS of the whole thoracic aorta from 4D flow MRI data.

## Methods

**Figure 1:**
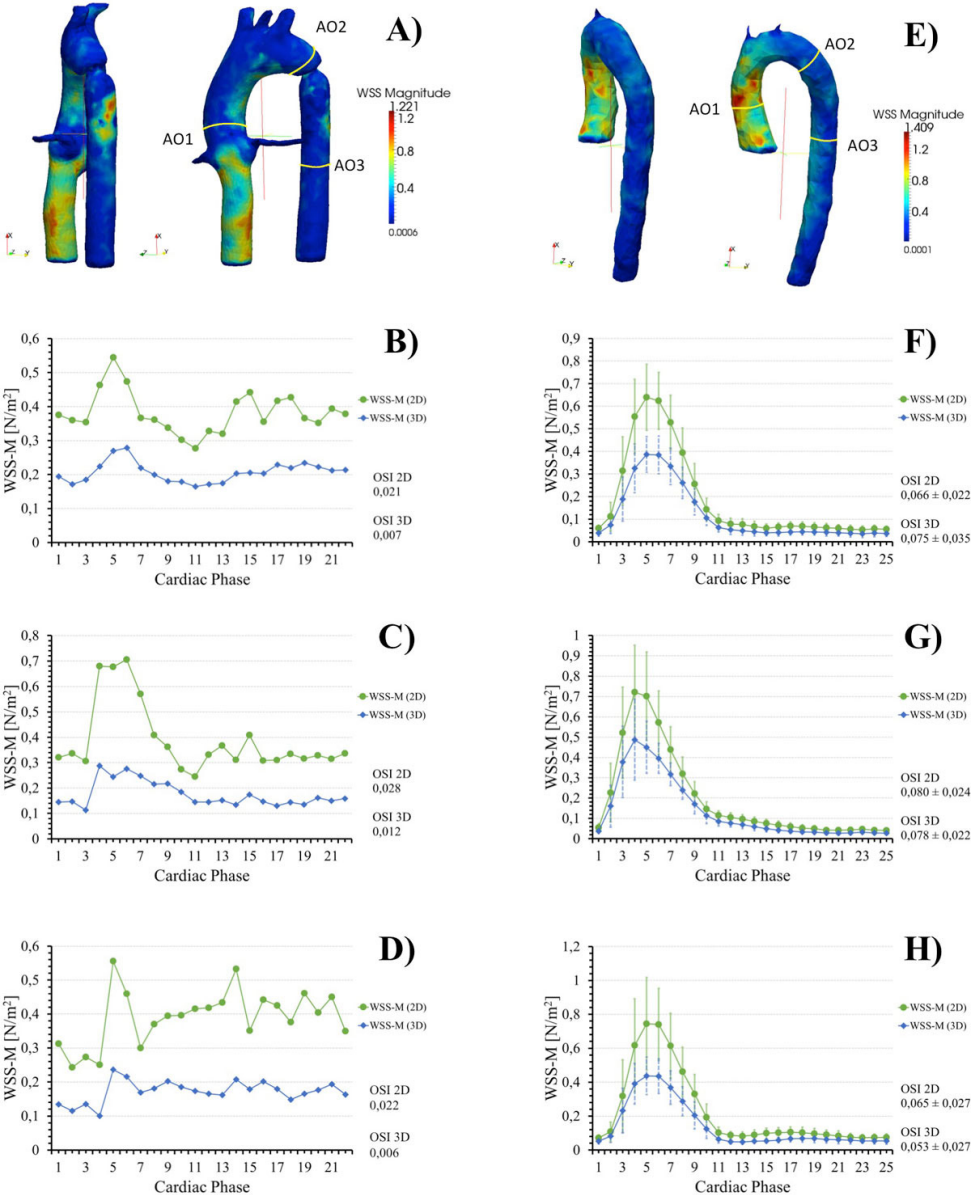
**The first row shows the WSS magnitude (WSS-M) in the entire thoracic aorta, obtained from a Phantom that simulates an aortic coarctation of 9 mm (A) and from one Volunteer (E)**. The WSS-M and OSI contour mean for each 2D cutting plane and standard across volunteers are depicted in B, F (AO1), C, G (AO2) D, H (AO3). It can be notice that the 3D and 2D WSS have the same shape distribution although the 2D WSS had in general larger values.

The entire aorta is segmented and then discretized using tetrahedral elements. The velocities at the each node are interpolated from 4D flow data using a cubic approximation. The shear stress tensor is calculated from the global least-squares stress-projection method, which is used to obtain the WSS. We compute the WSS distribution in the whole aortic vessel for 15 healthy volunteers and for a phantom that emulates an aortic coarctation. To showcase the applicability of our method, we report and compare the WSS in three 2D cutting planes of the aorta along the cardiac cycle. We compare reformatted 2D WSS data from the 3D WSS and 2D WSS calculated over reformatted 2D planes from 4D flow data. The sections analyzed are: AO1 = ascending aorta, AO2 = final of aortic arch, AO3 = descending aorta as can be seen in Figure 1-A or 1-E.

## Results

**Figure 2:**
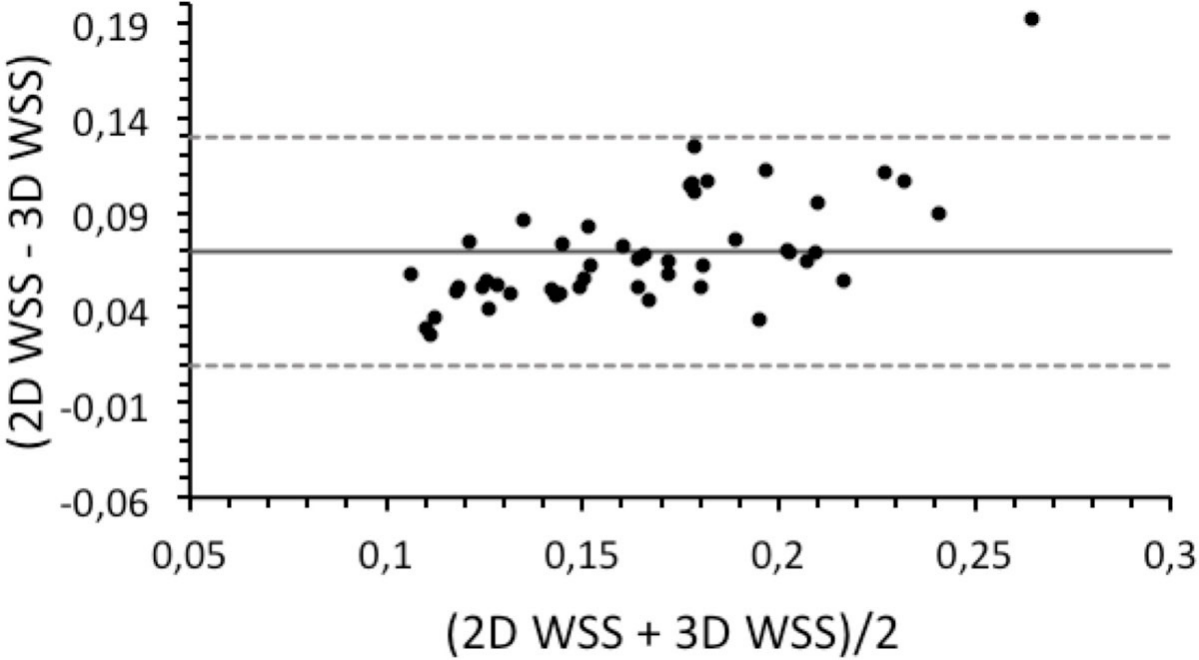
**Bland-Altman plot of cardiac phase averaged WSS contour mean comparing the 3D WSS and the 2D WSS method obtained from volunteer data for the three 2D sections of the Aorta**.

Our results showed that the magnitude of WSS contour mean values were in good agreement with the 2D approximation values obtained from 4D flow data (Figure 1). By observing the 3D WSS distribution in the entire aorta, it is easy to detect areas with high or low WSS, which is difficult to see with 2D methods (Figure 1 A-E). The mean and standard deviation of WSS contour mean values were lower for the 3D method than the 2D method in volunteers (Figure 1 F-G-H). We found that the OSI contour mean values in volunteers obtained by our method were lower than the 2D method in the sections AO1 and AO3 as shown in Figure 1. The Bland-Altman plot (Figure 2) showed a systematic bias between both methods with an average WSS contour mean difference of 0.069 ± 0.03 N/m^2^.

## Conclusions

To the best of our knowledge, this is the first report that presents a methodology to calculate WSS from 3D FE interpolations of the 4D flow data taken from the thoracic aorta.

## Funding

VRI # 44/2011 (Pontificia Universidad Católica de Chile), Anillo ACT 079 and FONDECYT #11100427 and #11121224. JS thanks CONICYT for scholarship for doctoral studies.

